# Skiing and Thinking About It: Moment-to-Moment and Retrospective Analysis of Emotions in an Extreme Sport

**DOI:** 10.3389/fpsyg.2018.00971

**Published:** 2018-06-20

**Authors:** Audun Hetland, Joar Vittersø, Simen Oscar Bø Wie, Eirik Kjelstrup, Matthias Mittner, Tove Irene Dahl

**Affiliations:** Department of Psychology, UiT The Arctic University of Norway, Tromsø, Norway

**Keywords:** emotions, facial expression, moment-to-moment, functional wellbeing approach, extreme sport, backcountry skiing

## Abstract

Happiness is typically reported as an important reason for participating in challenging activities like extreme sport. While in the middle of the activity, however, participants do not seem particularly happy. So where does the happiness come from? The article proposes some answers from a study of facially expressed emotions measured moment-by-moment during a backcountry skiing event. Self-reported emotions were also assessed immediately after the skiing. Participants expressed lower levels of happiness while skiing, compared to when stopping for a break. Moment-to-moment and self-reported measures of emotions were largely unrelated. These findings are explained with reference to the Functional Wellbeing Approach (Vittersø, 2013), which argues that some moment-to-moment feelings are non-evaluative in the sense of being generated directly by the difficulty of an activity. By contrast, retrospective emotional feelings are more complex as they include an evaluation of the overall goals and values associated with the activity as a whole.

## Introduction

We engage in recreational activities in order to feel good. This pursuit is not restricted to leisure activities like sunbathing at the beach or enjoying a fine meal with friends and family. Mountaineers, BASE jumpers, and other extreme athletes also claim that the importance of their favorite activities is the experience of positive feelings (Brymer, 2005; Willig, 2008; Brown and Fraser, 2009; Hetland and Vittersø, 2012). But what exactly is it that feels so good about these vigorous and exhausting activities, often referred to as extreme sport? To explore this question, we developed a new way of measuring emotions in real time during the activity. We equipped the participants with a camera that captured their facially expressed emotion while skiing. These films were then analyzed with software for automatic coding of facial expressions and compared the participants self-reported emotions assessed in retrospect. This approach enabled us to explore long standing questions as to how such positive experiences are created. Are they a result of a series of online positive feelings? Or is it the impact of a few central features like intensity peaks, rapid emotional changes, and happy endings that create them? Is it the experience of flow? Or is it the feeling of mastery that kicks in only after the activity has been successfully accomplished?

The present study explores these questions in order to enhance our understanding of the somewhat puzzling reports from extreme sport athletes about feeling good while performing activities like backcountry skiing, that appear—on the surface—to be extremely strenuous and highly unpleasant. We start with a brief clarification of the terminology regarding extreme sport.

### Extreme Sport

The term extreme sports covers a wide range of mostly individualized activities which are becoming increasingly popular in the western world (Thorpe and Wheaton, 2013; Brymer and Houge Mackenzie, 2017). Despite the significant growth in participants, media coverage, and research, it seems difficult to reach a clear-cut definition of what extreme sport is, or which activities fall under its umbrella. The term is often used interchangeably with other terms like action sport, adventure sport, lifestyle sport, alternative sport, and also adventure tourism (Puchan, 2004; Brymer and Schweitzer, 2013).

The label extreme sport is often defined as recreational physical activities that carry a risk of serious physical injury or even death (Willig, 2008; Haegeli and Pröbstl-Haider, 2016; Brymer and Schweitzer, 2017). This definition includes activities like BASE jumping, skydiving, hang gliding and paragliding, mountain climbing, surfing, white water kayaking, mountain biking, and backcountry skiing. However, Brown and Fraser (2009) argue that there has been a misconception in the literature. Even though many of these activities inherently involve some level of risk, risk itself is not a main motive. Woodman et al. (2010) argue that there is a considerable diversity among these activities across a range of measures. For example, Barlow et al. (2013) demonstrated that skydiving is strongly associated with sensation seeking, mountaineering on the other hand is not associated with sensation seeking, but rather emotion regulation and agency.

However, even though the activities under this umbrella differ substantially, they share one important feature. These activities allow the participants to create an optimal level of challenge where they are able to stretch themselves toward the edge of their skills while simultaneously insisting on doing so within the limits of their capabilities (Celsi et al., 1993; Larkin and Griffiths, 2004; Willig, 2008; Brymer and Gray, 2009; Kerr and Houge Mackenzie, 2012; Brymer and Schweitzer, 2013). According to Willig (2008), the motivation seems to be twofold. First, it includes a rational and functional motive where participants seek to increase their levels of skill and mastery. Second, participants are motivated to repeat activities that produce positive emotions.

### The Feeling of Skill Development

Most skills will not develop unless they are practiced. However the psychological literature diverges when it comes to how such training is experienced. One tradition, going back to Aristotle, will have it that skill development feels good if executed according to certain principles. Different versions of this approach are found in the Eudaimonic identity theory (Waterman, 2008) and flow theory (Csikszentmihalyi, 2009) Self-determination theory (Deci, 1975; Deci and Ryan, 1985, 2000) and the Functional wellbeing approach (Vittersø, 2013, 2016, 2018). The competing position argues that the development of talent requires considerable concentration and effort, and consequently, that the training of skills does not feel good. Ericsson and his colleague are proponents of this view (Ericsson et al., 1993).

Ericsson and his colleagues argue that skills do not develop from enjoyment, but from deliberate practice (Ericsson et al., 1993). Not unlike the preconditions for flow, deliberate practice requires clear feedback, possibility for repetition, correction of errors, and a task at an appropriate level of difficulty. However, in contrast to flow, Ericsson argues that this process of developing skills into excellence requires considerable efforts and is not inherently enjoyable.

This assumption is supported by several studies that demonstrate that flow is not an optimal experience, at least not in the sense of being enjoyable, and, at the same time contributing to the development of skills. Schüler and Brunner (2009) for example, found that during a marathon race, flow was not associated with better performance. Another study compared amateur and professional singers and found that the amateurs put less effort into their rehearsals and reported more enjoyment while singing (Grape et al., 2002).

Eudaimonic identity theory suggests that people typically feel good when their true potential is realized. According to Waterman (2008) these experiences are often described as “feeling really alive” or “feeling fulfilled.” Flow theory also suggests a link between optimal experience and optimal functioning where “the person feels simultaneously cognitively efficient, motivated and happy” (Moneta and Csikszentmihalyi, 1996, p. 277). In order to experience a state of flow, three conditions are especially important. These are: clear goals, immediate feedback, and a balance between perceived challenge and perceived skill (Csikszentmihalyi, 2009, pp. 397–398). If these are present, they might lead the actor to experience a merging of action and awareness, a sense of control, an altered sense of time and loss of self-consciousness.

Self-determination theory (SDT; Deci, 1975; Deci and Ryan, 1985, 2000; Ryan and Deci, 2017) is a widely cited theory about human behavior and personal development. Grounded in an organismic approach to the understanding of human nature, the SDT claims that humans are intrinsically motivated by “default.” Unless interrupted by an external motivation or a wish to experience strong, positive affect, humans will continuously do things they expect will satisfy three basic psychological needs. The needs are feeling competent, feeling self-determined (autonomous) and feeling related. People will typically experience these feelings in challenging situations, and that is why they often seek out situations that are challenging. According to SDT then, an activity such as backcountry skiing will be initiated by persons who expect that this activity will provide them with feelings of competence, self-determination and relatedness. When these feelings are experienced, their basic psychological needs are fulfilled.

Hence, SDT offers an explanation of why people engage in behavior such as backcountry skiing, but not of how feelings in and by themselves can be motivating. Neither does it provide a systematic account of feelings other than those related to competence, autonomy and relatedness (including the satisfaction that these feelings elicit), nor any details about the structure and functions of such feelings.

The Functional wellbeing approach (FWA; Vittersø, 2013, 2016, 2018) offers such an account, and a brief resume will be given below. The interested reader is referred to Vittersø (2018) for further details.

In order to explain the structural, functional and motivational qualities of activity-related feelings, FWA draws on three major strands of research. One is from Piaget and his concept of functional meaning (e.g., Piaget, 1952) What Piaget meant by functional meaning was, in short, that being active is an innate predisposition in any living organism. A biological structure, by virtue of being alive, encompasses the inclination to exercise its own structure. To operate is part of the structure itself and no further mechanism, like a need or other “energizers,” is required to explain why it becomes active.

The second major influence comes from Scheme theory (Eckblad, 1981) which offers a comprehensive account of intrinsic motivation. It is grounded in the work of Piaget, including his notion of the novelty principle. Piaget’s novelty principle states that humans are typically attracted to stimuli and environments that are somewhat novel and complex. In terms of cognitive processing, such environments will not be easily assimilated into existing schemas—rather, they *resist* assimilation. Eckblad took this idea one step further, by combining it with the Wundt curve, i.e., the idea that pleasure is related to arousal in a curvilinear manner. The two principles enabled Eckblad to develop a multi-curve model in which distinct feelings reached their most intense expressions at increasingly higher levels of assimilation resistance. Thus, each feeling state has “a single-peaked preference function for any given complexity dimension” (Eckblad, 1981, p. 97). Accordingly, when we are in a familiar and low-complex environment, our feelings will typically be experienced as calm, pleasant, and satisfactory. As the level of assimilation resistance increases, the qualities of the feelings will change toward happiness and joy. The experiences generated by even higher levels of assimilation resistance include feelings of interestingness and challenge. Still higher levels of assimilation resistance produce feelings like frustration or fear, and at that point, the activity is no longer intrinsically motivated.

The third relevant element of the FWA comes from mainstream emotions theories such as the Communicative Theory of Emotions (Oatley and Johnson-laird, 1987; Oatley, 1992) and appraisal theories (Smith and Ellsworth, 1985; Lazarus, 1991; Scherer et al., 2001). These theories hold the view that emotions are evaluative responses to challenges or opportunities regarding goals that are important to us. These appraisal-based emotions will typically occur in conjunction with evaluations of goal status. For instance, happiness will be felt when a goal or a sub-goal is reached, whereas sadness occurs when an important goal (i.e., something valuable) is lost, or when a major plan has failed. The FWA accepts these explanatory principle of emotions, but it suggests that a different kind of feeling exists, as well those generated according to the principles laid out in Eckblad’s multi-curve model.

### Moment-to-Moment vs. Retrospective Emotions

Early emotion theorists did not make a sharp distinction between moment-to-moment feelings and the memory of these feelings, and retrospective happiness was seen as nothing but accumulation of moment-to-moment pleasures (e.g., Edgeworth, 1881). Happiness in a certain time interval could therefore be quantified as the temporal integral of the experienced pleasure (net of pain) during that event or episode. Despite its controversial content, respected scholars have adopted the essence of this idea (e.g., Cabanac, 1992; Parducci, 1995; Kahneman et al., 1997; Kahneman, 1999).

Although supportive of the idea that feelings can be measured on a moment-by-moment basis, Kahneman has never argued that a mere adding up of such on-line feelings is equivalent to the mental image we store in memory to represent the emotional quality of the time interval in question. Rather, he introduced a distinction between moment-to-moment feelings, or the experiencing self, and memories of that feeling—the remembering self. In an impressive series of studies, Kahneman and his colleagues have documented how remembered feelings do not amount to the sum of the moment-by-moment experiences during an event or episode (Kahneman et al., 1993; Kahneman, 1999; Schwarz et al., 2009; Kahneman and Deaton, 2010).

These studies show that instead of an accumulation of moment-to-moment feelings, the memory of these feelings is created based on the emotional experience at certain key points like beginning, end and emotional peaks during the experience. Features like duration of the experience have been found to have little or no impact on the retrospective evaluations (Fredrickson and Kahneman, 1993). In line with relevant literature, we refer to these key point as gestalt characteristics (Ariely and Carmon, 2003).

A different view on the discrepancy between moment-to-moment feelings and retrospective memories comes from research on flow (Csikszentmihalyi, 1975, 1999). The experience of flow is characterized by enjoyment. However, during intense experiences of immersed and concentrated attention, people may be so absorbed in what they are doing that they do not consciously register the stream of experiences that are created by the body’s feeling system. The feelings only surface after the experience is over.

In contrast to flow theory, the FWA argues that people do indeed have intense feelings during flow, but not of enjoyment. Rather, feelings like interest, engagement and immersion comprise the prototypical phenomenology of a flow experience. Emotional feelings such as happiness may come afterward. The reason is, according to the FWA, that moment-to-moment feelings are integrated in the executive part of an activity, whereas the emotional feeling is part of the evaluative part of the activity. The separation of activities into an executive part and an evaluative part owes to the idea of Test-Operate-Test-Exit (TOTE) sequences described by Miller et al. (1960).

In the TOTE model, an activity is characterized by rapid shifts between operating and evaluating the outcome of the operation against the goal for the activity. Following the FWA, both the operating phase and the evaluative phase generate feelings, but according to different mechanisms. The feelings produced by the operating phase are determined by how difficult the activity is. If the task is too easy, we feel bored. At somewhat higher levels of difficulty we feel pleased, and as the difficulty level increases we feel interested and challenged, until the difficulty become too overwhelming, in which case the feelings become negative, like frustration, anger or fear. The idea about how difficulty and feeling states are associated is adopted from Eckblad (1981).

In phases of evaluation, the prospect of goal achievement regulates the emotional qualities, in line with the principles laid out in the Communicative Theory of Emotions (Oatley and Johnson-laird, 1987; Johnson-laird and Oatley, 1989; Oatley, 1992). Here it is proposed that emotions are responses to challenges or opportunities regarding goals that are important to us. Sadness, for instance, will typically occur when a major plan fails or an important goal (i.e., something valuable) is lost. Happiness, on the other hand, will be felt when a goal or a sub-goal is reached.

The above account of how variations in perceived difficulty create moment-to-moment feelings of different phenomenological qualities on the one hand, and how such moment-to-moment feelings interact with evaluative emotions on the other, resembles an explanation offered by Buckley (2016).

Buckley’s work concerns the interplay between fear and thrills. Based on data from more than 4000 participants collected across a range of risk related outdoor activities and measured with different methods he has identified a pattern of subjective experiences referred to as a “sawtooth relation between fear and thrill.” Buckley’s analysis shows how perceived danger generates a focused awareness, and that this, in turn, may suppress fear and other emotions during phases of intense concentration.

The sequence of a high-risk activity then unfolds by moments of focused attention “during the action or event, then thrill, relief, or triumph afterward. The emotionless state persists only during the most intense concentration.” (Buckley, 2016, p. 1). The author also describes a curvilinear relation between perceived risk and thrill. Below a certain threshold of risk, thrill can occur without fear, but when the perceived risk increases, feelings of fear will kick in. As the experience of fear increases, so does the feeling of thrill. However, above some upper threshold thrill vanishes, whereas fear remains. Buckley also noted an adaptation effect, in that the highest levels of fear were reported by first time participants during unfamiliar activities, but the feeling decreased as the experienced increased.

In order to investigate the role of feelings in motivation and skill development, both the level of difficulty and the evaluation of goal status must be considered. An important aim for the present study was to explore how the two principles operate during highly challenging activities such as extreme sport. However, before we proceed to investigate this aim further, a note on terminology is in order.

The paper operates with three levels of time segments. We use the term *event* for the entire happening under investigation, i.e., from when the skiers start at the top of the mountain until they end at the parking lot. The term *episode* is used for the next level, where the event is divided into seven smaller parts, like (1) at the top, one minute before start, (2) the first part of the descent, (3) before half-way, (4) half-way down the mountain, (5) after half-way, (6) last part of the descent and (7) immediately after stopping. The final time segment is referred to as *moment-to-moment experiences* or *moment-by-moment experiences*, which are used synonymously. A moment-to-moment experience is an ongoing emotional experience captured in real time. Finally, we will refer to the phenomenological aspect of an emotion as a subjective experience or, as a synonym, a feeling state.

### Measuring Emotions

The most common way to measure emotions is through various types of self-reports. However, in addition to the obvious challenges of reporting one’s emotions while skiing, putting experiences into words demands at least some level of cognitive reflection. Thus, if the participants are totally immersed, reporting emotions will be impossible (Nilsen and Kaszniak, 2007).

Some people will even have a hard time identifying and describing their emotions regardless of their level of activity. Such difficulty is called alexithymia and a recent study show that this deficit is connected to greater risk-taking (Barlow et al., 2015).

Kahneman and Riis (2005) have argued that when properly validated, physiological measures like Electroencephalography (EEG), heart rate or skin conductance level would offer an uninterrupted moment-to-moment report of emotions. But, at least in the case of extreme sport which almost always involves physical activity, the activity itself will create a considerable amount of noise, rendering it impossible to disentangle the effect of psychological activity expressed physiologically, from the mere physical activity itself. In addition, there are issues of translating these somatic measures into what they really mean in terms of psychologically experienced emotions.

Systematic observations of facial expressions of emotions offer another source of information about subjective experiences. According to Matsumoto et al. (2008), facial expressions are (1) universal and reliable indicators of discrete emotions which (2) co-vary with subjective experiences and (3) are a part of a coherent package of emotional responses that include appraisals, physiological reactions, and other nonverbal behaviors and subsequent actions. There is strong support for at least six universal facial expressions communicating happiness, fear, sadness, disgust, anger and surprise (Ekman, 1993). However, new studies suggest that as many as eight different positive emotion are associated with distinctive expressive displays. In addition to happiness, these emotions are amusement, awe, contentment, interest, joy, love and pride (Campos et al., 2012).

Observing participants facially expressed emotions has previously been tested as a way of capturing extreme sport experiences. Buckley (2016) observed or collected films of more than 4000 participants while performing different risk related activities. The results revealed a substantial variation in emotions. Unfortunately, none of the raters were trained in coding facial expressed emotions, and the author were unable to conduct any fine-grained analysis of emotions. This paper follows a similar approach but relies on a sophisticated method for analyzing facially expressed emotions through automatic facial coding (AFC) software.

To perform a reliable measure of facial expressions, Ekman and Friesen (1978) developed the facial action coding system (FACS) which is a way of directly measuring movements of the face. FACS consists of 46 action units, each representing an independent motion of the face, which, in turn, is combined in various ways into distinct facial expressions. A happy facial expression is, for example, indicated by a raised chin, measured by action unit six (AU6) and lip corners pulled upward (AU12). A sad facial expression is, on the other hand, characterized by raised inner brows (AU1), lowered brows (AU4) and depressed lip corners (AU15). However, interest, as described by Campos and his colleagues, is described by the same initial two action units (AU1 and AU4). What distinguishes facially expressed sadness from interest is that instead of depressed lip corners, an interested facial expression sometimes involves compressed lips (AU24) and raised outer brows (AU2).

In the past, the analysis of facial expressions had to be done manually. However, with advances in technology, this can now be done electronically. AFC has several advantages and a few disadvantages compared to manual coding. First and foremost, automatic capturing, interpreting, and coding demands very little labor. In addition, different studies have shown that such digital analyses outperform non-expert coders, and are approximately as accurate as expert coders (Bartlett et al., 1999; Terzis et al., 2010; Lewinski et al., 2014). In a reliability study, Lewinski et al. (2014) found that FaceReader, the software used in this study, recognized 88% of the targeted emotions in both the *Warsaw Set of Emotional Facial Expression* (WSEFEP; Olszanowski et al., 2014) and the *Amsterdam Dynamic Facial Expression Set* (ADFES; van der Schalk et al., 2011). The corresponding number for human raters was 85%. For happiness, the average FaceReader recognition score for the two picture databases was 96%, the corresponding score for human raters was 89%.

On the downside, the methods for coding facial expression are still developing, particularly in how they distinguish the display of different positive emotions (see for example Campos et al., 2012). It takes time before enough pre-coded material is available for a potential update of the computer model used by the AFC programs. In addition, humans are able to code other movements and body postures that the software is not currently able to read. Therefore, happiness is so far the only positive emotion included in the automatic analyses of facial expressions.

Still, the advantages of automatic coding in most cases outweigh the cost, making this method increasingly popular. It has previously been used in a number of fields like emotion science (Bartlett et al., 1999; Chentsova-Dutton and Tsai, 2010), educational research (Terzis et al., 2012, 2013; Chiu et al., 2014), human-computer interaction (Cohen et al., 2003), consumer behavior (Garcia-Burgos and Zamora, 2013; Danner et al., 2014; de Wijk et al., 2014), user experience (Goldberg, 2014), clinical investigations of facial nerve grading in medicine (Dulguerov et al., 1999), monitoring pain (Lucey et al., 2012) and reaction to advertisement and commercial films (Teixeira et al., 2012; Hetland et al., 2015). With video cameras for capturing facial expressions becoming increasingly better and smaller, this method can now be applied in new areas of investigation like extreme sports.

In the current study, we equipped participants with cameras in order to capture their facially expressed emotions while they skied. In addition, we asked them to report the same emotions in a self-report questionnaire immediately after the trip was over.

### Aims of the Study

The overarching aims of this study are to test a new method of measuring emotions in the field and use this method to investigate (1) how moment-to-moment feelings unfold during an extreme sport event and (2) how they are related to the emotional self-report presented at the end of the event. Based on these aims, three hypothesis and two research questions were put forward.

Hypotheses:

(1) Given the difficulty involved in backcountry skiing, and the rewarding role played by hedonic feeling when goals and sub-goals have been achieved, we refer to the FWA and predict that participants will be happier when they stop skiing, than when they are actively downhill skiing.
(2) Following the FWA, we hypothesize that there will not necessarily be a strong relationship between the moment-by-moment emotions experienced during a difficult event and the emotions reported from the overall event. This is so because the two kinds of emotions are generated by different mechanisms.
(3) Following the principles presented above as the gestalt characteristics of remembered emotions, we hypothesize that moment-to-moment emotions at key points, like the beginning and the end will predict self-reported event emotions.

We also put forward two research questions, for which the theories presented above give no reason to deduce clear hypotheses.

(1) What is the relationship between facially expressed emotions and the self-reported emotions for the 7 episodes of the event?
(2) What are the similarities and differences between pleasure, interest, and fear across the 7 episodes?

## Materials and Method

### Participants

Fifty-three backcountry skiers (34 men and 21 women) were recruited through social media and snowball sampling. Age ranged from 19 to 46 (*M* = 27.51, *SD* = 5.7). The large majority of the sample was Norwegian (*n* = 50) but also included skiers from Sweden (*n* = 2), United States (*n* = 1), Canada (*n* = 1) and Germany (*n* = 1).

### Procedure and Materials

The data for this study came from six different sources: Three self-report questionnaires, a computer-based analysis of facial expressions, heart rate measures and speed measures recorded during a skiing event. The questionnaires assessed (1) background variables (questionnaire A); (2) state emotions immediately after the trip (questionnaire B). A subsample also received (3) a follow up questionnaire 1 week after the trip (questionnaire C) assessing their memory of the trip and appurtenant state emotions. These questionnaires were available in Norwegian and English and were translated and back-translated to ensure equivalence across languages and cultures.

Moment-to-moment emotions were measured with (4) software that analyzed facial expressions. In addition, we also recorded the participants (5) heart rate (HR) and (6) speed.

In the present article, we have focused on data collected during the event (facially expressed emotions) and self-reported emotions immediately after the trip (questionnaire B). In addition, we have used GPS speed to code when the participants are skiing or taking a break.

On the day of skiing, the participants met the researchers in a parking lot at the foot of the mountain to be skied that day. The participants had freely chosen which mountain to go to, which route to take and run to ski. After signing an informed consent form, the participants were each given a ski-helmet. There were five helmets available with two cameras each.

The mounting for the camera is constructed such that it will not swing down and hit the participants face, but rather swing to the side, up or fall of. We demonstrated this feature to the participants and they all got to study the helmets before start of the trip and could wear them on their way up – or take short trial runs if they liked. They were of course also free to remove the camera at any time if they liked, but nobody did. We also asked the participants immediately after the trip about their experiences of skiing with this camera. All participants, with no exception, reported that they habituated quickly and for the most forgot about the camera.

The first camera was attached to the helmet by the skier’s ear in order to film the descent from the skier’s point of view. The other camera was mounted in front of the skier’s face in order to capture his or her facial expressions during the ski (see image below). Each participant was also equipped with a heart rate monitor that recorded speed based on GPS coordinates. At least one researcher accompanied the participants the whole trip. Before they started hiking up, the researchers explained that they would not state any opinion on matters concerning route planning or avalanche safety, though they would take a different route than the skiers if it was determined that the chosen route appeared too dangerous. Aside from that, the researchers interacted with the participants during the whole trip as another member of the ski party. The region where these data were collected has a vast number of mountains typically ranging from 800 to 1200 meters above sea level with very little forest. The typical accent/decent was 600–800 meters of altitude gain/loss.

At the top of the mountain, the participants geared up with cameras and heart rate monitors. The researchers synchronized the two cameras and HR monitor for each skier. The participants then skied at their own pace down the mountain. Immediately after the skiers returned to the parking lot, the researchers distributed a second questionnaire (questionnaire B) that the skiers filled out immediately.

### Facially Expressed Emotion

#### The Camera

**FIGURE 1:**
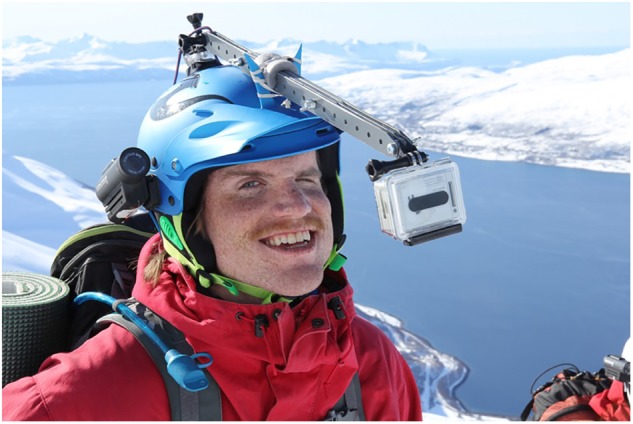
The helmet and cameras used for collecting data. Written informed consent has been obtained from the participant for the publication of this image.

The facially expressed emotions were captured with GoPro 4 cameras set to full HD (1080) progressive, a frame rate of 25 images pr. second and field of view (FOV) set to medium. Based on these settings the film consists of 25 self-contained still images pr. second where the entire face of the participants was visible. **Figure 1** shows how the camera was mounted on the helmet.

#### The Technique

The film was then analyzed using FaceReader v5.0 (Noldus, 2014a). This computer software bases its interpretations and coding’s on the FACS (Ekman and Friesen, 1978) for scoring facial expressions. To be able to automatically read facial expressions, the software creates a 3D model of a human face. This model consists of a mesh with 491 measuring points that uses an algorithmic approach to code movements based on the active appearance method (Cootes and Taylor, 2004). The model has been trained on approximately 10,000 images scored by certified FACS experts.

The raw data from FaceReader were then imported to Observer XT v12 (Noldus, 2014b). The data were thereafter synchronized with the films from the two cameras as well as the heart rate and speed data. Based on the two camera angels and speed data, each participant’s behavior was coded into skiing and breaks. Breaks were defined to be complete stops of at least a 10-s duration or times when the participants moved so slowly that they needed to use their ski poles to move forward.

#### The Variables

The AFC software detects the face, applies and adapts the mesh to the individual’s features, and by measuring the ongoing movement between the points, it automatically classifies facial motion into the six basic emotions of happy, sad, angry, disgust, fear and surprise, all scored on a scale from 0 to 1. In addition, a neutral state is also recorded. These emotions are not mutually exclusive, meaning that several emotions may register at different magnitudes at the same time. The film was analyzed frame by frame, providing us with 25 measures every second that captured the intensity and duration of each of the six basic emotions and the neutral state during the whole descent.

### Self-Reported Emotions

#### Self-Reported Emotions for the Entire Event

Immediately after the downhill run was completed, the participants reported on their experience of five basic emotions. We refer to these measures as self-reported event emotions. The five emotions were (1) happiness (measured by the three adjectives happiness, pleasure and satisfaction), (2) interest (measured by the three adjectives interest, engagement, and enthusiasm), (3) fear, (4) anger, and (5) sadness. Each of the three negative emotions were measured with one item each (the adjectives fear, anger and sadness). The items were presented with the introduction: “Now, let us look at your total experience of skiing down the mountain. There are a number of emotions you may have experienced, to a varying extent. Try to recall how you felt while you were skiing, and check the number that best describes your emotions. Note that you have to answer the first nine questions to be able to proceed.” For each item, the participants responded using an end-point labeled rating scale ranging from 1 (Not at all) to 7 (Very much).

Because the main interest of the present work was toward positive feelings, we sampled these with multi-item scales. The three happiness adjectives were collapsed into a mean-score happiness variable [Cronbach’s alpha (α) = 0.91]. The three interest adjectives were lumped into a mean-score interest variable (α = 0.79). When the six items were fitted to a two-factor confirmatory factor analytic model (CFA), an acceptable fit was returned, χ^2^(8, *N* = 36) = 14. 59, *p* = 0.068. This chi-square was a significantly better than that from a one-factor model Δχ^2^(1) = 5.74, *p* = 0.017. As a kind of robustness test of the two-factor structure for positive emotions, an additional CFA was performed on six emotion items from the European Social Survey (*N* = 42648). We used data from wave 6, since it contains a wellbeing module that includes emotion questions about happiness, enjoyment and calmness (as indicators of happiness) and items on interestingness, absorption and enthusiasm (as indicators of interest). Again, the CFA supports the notion that these six items belong to two different dimensions of positive emotions. The difference in goodness-of fit between the one-factor model and the two-factor model was highly significant, Δχ^2^ (1) = 27024, *p* < 0.001.

#### Self-Reported Emotions for the 7 Episodes

Self-reported episode emotions were also measured immediately after the activity. The participants reported their level of pleasure, interest, and fear at seven different stages of the descent, from (1) at the top, one minute before start, (2) the first part of the descent, (3) before half-way, (4) half-way down the mountain, (5) after half-way, (6) last part of the descent and (7) immediately after stopping. The items were presented with the introduction “Below you will find a graph with a time line (horizontal) and a “feelometer” scale on the vertical axis, ranging from 0 (not pleasant/ not interesting/no fear) to 10 (very pleasant/ very interesting/ very much fear). We want you to state how much (pleasure/interest/fear) you experienced at the different parts of the ski trip from when you stood at the top until you stopped at the foot of the mountain. To the left you will see a short description of the different parts of the trip. Please answer by clicking on the horizontal graph on each question.

#### Data Quality and Missing Data

The data quality varied across participants and during the event. This is partly due to the fact that the recruited skiers represented a wide range of skill levels (resulting in faster or slower descents) and that the data were gathered on a range of different mountains (resulting in longer or shorter routes).

Factors influencing data-quality were frequency of falls, sun in the background (which made the automatic identification of facial expressions impossible), accidental changes in camera position (e.g., after a fall) or changes in face visibility (e.g., scarves that were adjusted and then occluded parts of the face).

Regarding the very different percentages of missing values, we have to specify exclusion criteria for each of the analyses separately. These are specified and discussed for each analysis as the available data pose restrictions in different ways for different analyses.

## Results

### Descriptive Statistics

**FIGURE 2:**
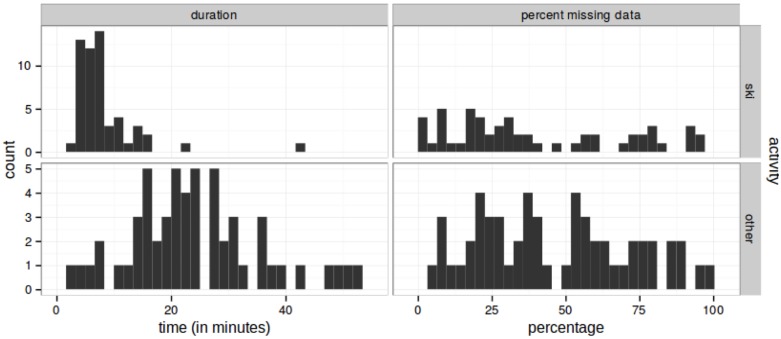
Total duration (in minutes, left column) and missing data (in percent, right column) of skiing (top row) and ski-breaks (bottom row). Written informed consent has been obtained from the participant for the publication of this image.

The mean ski trip duration was 32.49 min (ranging from 7.96 to 92.69 min with *SD* = 15.08). **Figure 2** shows histograms of the total duration (number of minutes, left panel) that our participants were skiing (top row) or not skiing (bottom row and labeled “other”) and the percentage of missing data points (right column). The data quality varied across participants and over the event, resulting in a mean percentage of missing data of 44.50%, *SD* = 25.50). For descriptive statistics for self-reported emotions see **Table 1**.

**Table 1:** Descriptive statistics with mean (M) standard deviation (SD) and standard error of mean (SEM) for self-reported episode emotions.

Variable	*M*	*SD*	*SEM*
Pleasure	5.34	1.80	0.83
Happiness	5.76	1.34	0.90
Satisfaction	5.63	1.55	0.88
Interest	5.12	1.38	0.80
Engagement	4.63	1.61	0.72
Enthusiasm	5.10	1.39	0.80
Fear	2.46	1.66	0.39
Anger	1.68	1.51	0.26
Sadness	1.34	1.24	0.21

*N = 43*.

### Difficult Activities Are Not Pleasant

The first hypothesis predicts that participants are happier when they stop skiing, than when they are actively downhill skiing, and facially expressed happiness was used to test it. Only participants with at least three minutes of valid skiing data and another 3 min of data collected in the breaks were included in the test of our first hypothesis, providing a sample of *N* = 33 for this analysis. This sample consisted of 20 men and 13 women with an age range from 19 to 36 years (*M* = 25.76, *SD* = 4.07).

A linear mixed model with random intercepts and emotions (with neutral as the baseline) and activity type – ski or break (with skiing as the baseline) as regressors was conducted. The *t*-tests used the Satterthwaite approximation with *df* = 2.

**FIGURE 3:**
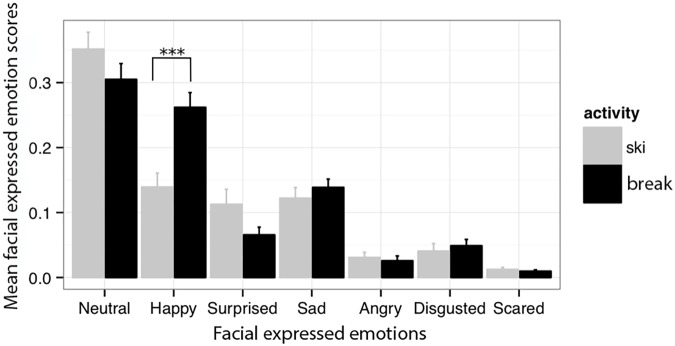
Mean facial expressed emotions during skiing (gray) and breaks (black) across the six facial expressed emotions plus the generated neutral state. ^∗∗∗^*p* < 0.001.

A main effect for activity was found, with an unstandardized regression coefficient *B* = 0.05, *p* = 0.043 indicating that facially expressed emotions were more intense during skiing than during ski breaks. In addition, all five emotions showed significantly lower activation scores compared to the “Neutral” classification (all *p*s < 001). More interestingly, happy feelings increased significantly during breaks (baseline), *B* = 0.169, *p* < 0.001, confirming Hypothesis 1. Please cf. **Figure 3** and **Table 2** for further details.

**Table 2:** Summary of the random intercepts model for mean (M) and standard error of the mean (SEM) and goodness of fits indicators for facially expressed emotions during skiing and breaks.

	*Dependent variable* Mean facially expressed emotion	
	*M*	*SEM*
Happy	-0.21^∗∗∗^	(0.02)
Surprised	-0.24^∗∗∗^	(0.02)
Sad	-0.23^∗∗∗^	(0.02)
Angry	-0.32^∗∗∗^	(0.02)
Disgusted	-0.31^∗∗∗^	(0.02)
Scared	-0.34^∗∗∗^	(0.02)
ski-breaks × Happy	0.17^∗∗∗^	(0.03)
ski-breaks × Surprised	-0.00	(0.03)
ski-breaks × Sad	0.06	(0.03)
ski-breaks × Angry	0.04	(0.03)
ski-breaks × Disgusted	0.06	(0.03)
ski-breaks × Scared	0.04	(0.03)
ski – breaks	-0.00	
Intercept	0.35^∗∗∗^	(0.02)
Log Likelihood	402.245	
Akaike Inf. Crit.	-772.49	
Bayesian Inf. Crit.	-706.321	

^∗∗∗^*p < 0.001*.

### Moment-by-Moment Emotions and Recalled Event Emotions

In accordance with the FWA, the second hypothesis predicts that there will not be a strong relationship between the moment-by-moment emotions experienced during a difficult event and the emotions reported from the overall event.

To test this hypothesis we ran two series of independent linear regression analyses. In the first series, we used the total means of the seven facially expressed event emotions as independent variables in five separate regression models (one for each of the self-reported emotions). Only participants with at least three minutes of valid data from skiing and completed self-reported event scores were included in the first analyses, providing a sample of 46 participants (27 male, 19 female, age range 19–46, *M* = 26.96, *SD* = 5.51).

The results from these five analyses are reported in **Table 3**. As can be seen in the table, only few of the facially expressed event emotions predicted self-reported event emotions: Facially expressed sadness was inversely related to self-reported happiness (β = -0.56, *p* = 0.020) and facially expressed fear was related to self-reported interest (β = 0.37, *p* = 0.041). In addition, neutral expressions predicted self-reported anger (β = 0.57, *p* = 0.022) and sadness (β = 0.69, *p* = 0.0027).

**Table 3:** Standardized regression coefficients (β), standard errors (in parenthesis) and overall statistics for five models predicting self-reported event emotions by the means of the facially expressed emotions.

	Self-reported event emotions				
Mean facially expressed emotions	Anger	Fear	Sadness	Happiness	Interest
Neutral	0.57^∗^ (0.24)	0.36 (0.23)	0.69^∗^ (0.22)	-0.24 (0.24)	-0.06 (0.23)
Happy	0.21 (0.25)	0.01 (0.24)	0.11 (0.22)	-0.05 (0.25)	0.24 (0.24)
Surprised	0.01 (0.23)	-0.02 (0.22)	0.06 (0.20)	-0.37 (0.23)	-0.10 (0.22)
Sad	0.33 (0.23)	0.32 (0.22)	0.10 (0.21)	-0.57^∗^ (0.23)	-0.29 (0.22)
Angry	0.07 (0.18)	-0.13 (0.17)	-0.02 (0.16)	-0.05 (0.18)	-0.17 (0.18)
Disgusted	-0.03 (0.19)	-0.23 (0.18)	0.01 (0.17)	0.10 (0.19)	0.27 (0.18)
Scared	0.17 (0.18)	0.21 (0.18)	0.01 (0.16)	0.30 (0.18)	0.37^∗^ (0.18)
Intercept	0.00 (0.14)	0.00 (0.14)	-0.00 (0.13)	0.00 (0.14)	0.00 (0.14)

*R*^2^	0.22	0.27	0.37	0.22	0.27
Adjusted *R*^2^	0.08	0.14	0.26	0.08	0.13
Residual Std. Error (*df* = 38)	0.96	0.93	0.87	0.96	0.93
F Statistic (*df* = 7; 38)	1.56	2.03	3.24^∗∗^	1.57	1.98
*p*-values	0.170	0.077	0.009	0.174	0.084

^∗^*p < 0.05; ^∗∗^*p <* 0.01, *N* = 46*.

In the second series of analyses, we divided the facially expressed emotions into skiing and breaks. Only participants with at least three minutes of valid data in each of the categories (skiing and breaks) and completed self-reported event scores were included, providing a sample of 31 participants (18 male, 13 female, age range 19–36, *M* = 25.74, *SD* = 4.17).

Neither of the facially expressed emotions for skiing nor the facially expressed emotions for breaks predicted self-reported event emotions. The results are summarized in **Table 4**.

**Table 4:** Standardized regression coefficients (β), standard errors (in parenthesis) and overall statistics for five models predicting self-reported event emotions by the means of the facially expressed emotions during breaks (upper part) and skiing (lower part).

	Overall self-reported emotions				
Facially expressed emotions	Anger	Fear	Sadness	Happiness	Interest
**During break**					
Neutral	-0.01 (0.76)	-0.61 (0.78)	0.19 (0.76)	-0.42 (0.70)	-0.67 (0.73)
Happy	-0.56 (0.67)	-0.73 (0.68)	0.11 (0.66)	-0.20 (0.61)	-0.22 (0.64)
Surprised	-0.48 (0.75)	-0.18 (0.76)	0.48 (0.74)	-0.44 (0.68)	0.30 (0.72)
Sad	-0.44 (0.51)	0.09 (0.51)	0.14 (0.50)	-0.85 (0.46)	-0.27 (0.49)
Angry	-0.33 (0.54)	-0.55 (0.54)	-0.06 (0.53)	0.46 (0.49)	0.21 (0.51)
Disgusted	0.74 (0.46)	-0.16 (0.46)	0.07 (0.45)	-0.07 (0.42)	-0.14 (0.44)
Scared	-0.28 (0.44)	-0.22 (0.45)	-0.20 (0.44)	0.24 (0.41)	-0.11 (0.43)
**During ski**					
Neutral	0.80 (0.71)	0.84 (0.72)	0.84 (0.71)	-0.11 (0.65)	0.49 (0.68)
Happy	0.84 (0.65)	0.71 (0.66)	0.27 (0.64)	-0.13 (0.59)	0.44 (0.62)
Surprised	0.44 (0.47)	-0.13 (0.48)	-0.01 (0.47)	-0.53 (0.43)	-0.71 (0.45)
Sad	0.67 (0.38)	0.05 (0.39)	0.29 (0.38)	0.02 (0.35)	-0.14 (0.36)
Angry	0.79 (0.58)	0.39 (0.59)	0.03 (0.58)	-0.46 (0.53)	-0.29 (0.56)
Disgusted	-0.83 (0.49)	-0.11 (0.50)	-0.01 (0.48)	0.24 (0.45)	0.34 (0.47)
Scared	0.61 (0.44)	0.51 (0.44)	-0.12 (0.43)	0.30 (0.40)	0.43 (0.42)

Intercept	-0.00 (0.19)	0.00 (0.19)	0.00 (0.18)	-0.00 (0.17)	-0.00 (0.18)

*R*^2^	0.44	0.42	0.44	0.53	0.48
Adjusted *R*^2^	-0.06	-0.09	-0.04	0.11	0.03
Residual Std. Error (*df* = 16)	1.03	1.04	1.02	0.94	0.99
F Statistic (*df* = 14; 16)	0.86	0.83	0.91	1.27	1.06
*p*-values	0.580	0.630	0.560	0.320^∗^	0.450^∗^

^∗^*p < 0.05, *N* = 31*.

Both series of analyses went against the predictions offered by the hedonic approach and in favor of the predictions of the FWA. These results are in line with the second hypothesis.

### Gestalt Characteristics of the Moment-by-Moment Emotions at the Event Level

**FIGURE 4:**
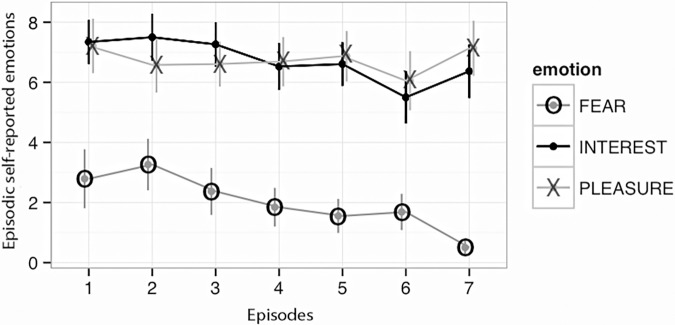
Mean self-reported episodic fear, interest and pleasure for the seven episodes.

The third hypothesis predicts that self-reported event emotions can be predicted by certain gestalt characteristics of the moment-by-moment emotions, such as the emotions at the beginning or the end of the event. Facially expressed emotions were used as predictors to test the hypothesis. First, the data from the facially expressed emotion were divided into seven equal episodes, corresponding to the self-reported episodic emotions, as displayed in **Figure 4** (cf. see section “Materials and Methods”), hereafter referred to as facially expressed episodic emotions.

Only participants with a minimum of 30 s of valid facial data in each of the seven episodes and with a complete record of self-reported event emotions were included, resulting in a sample of *N* = 27 participants (16 male, 11 female, age range 19–39, *M* = 26.44, *SD* = 4.74).

The results showed that none of the facially expressed emotions in any of these different episodes significantly predicted overall self-reported emotions. There were, in other words, no beginning or end effects, so Hypothesis 3 was not confirmed.

### Moment-by-Moment and Recalled Emotions as Episodes

We also put forward two research questions, for which the presented theories gave no reason to deduce clear hypotheses. The first addresses the relation between the facially expressed episode emotions and the self-reported episode emotions.

We sat up three independent, random-intercept linear mixed models, with the same pre-conditions and sample as in the previous analysis. Each of the self-reported episode emotions (pleasure, interest and fear) was predicted by their corresponding facially expressed episode emotions. A new variable was generated to express the order of the episodes from 1 (the first episode) to 7 (the last episode). The new episode progress variable thus reflects the development of the three emotions during the entire trip.

To test whether the facially expressed episode emotions would add predictive power over and above what is to be expected from each of the episodes themselves, we compared the full model to a baseline model which had only episodes as a predictor (and random-intercepts). This comparison was not significant for self-reported pleasure and interest (pleasure: χ^2^(7) = 10.8, *p* = 0.149; interest: χ^2^(7) = 4.88, *p* = 0.674) but reached significance for self-reported fear, χ^2^(7) = 18.51, *p* = 0.010.

When investigating these models in more detail, however, we found that facially expressed episode sadness predicted self-reported episode fear, β = 0.68, *p* = 0.001 and facially expressed fear was inversely related to reported pleasure, β = -2.37, *p* = 0.049 (cf. **Table 5**).

**Table 5:** Standardized regression coefficients (β), standard errors (in parenthesis) and overall statistics for three models with self-reported episode emotions as dependent and facially expressed episode emotions as predictor variables.

	Self-reported episode emotions		
Facially expressed episode emotions	Pleasure	Interest	Fear
Happy	0.19 (0.20)	0.28 (0.19)	-0.00 (0.16)
Neutral	-0.20 (0.20)	0.23 (0.19)	0.16 (0.15)
Angry	-0.20 (0.55)	-0.10 (0.53)	-0.81 (0.42)
Disgusted	0.23 (0.32)	0.33 (0.31)	-0.22 (0.25)
Surprised	0.06 (0.21)	0.28 (0.20)	0.10 (0.16)
Scared	-0.37^∗^ (1.19)	-1.16 (1.14)	0.85 (0.93)
Sad	-0.19 (0.27)	0.20 (0.25)	0.68^∗∗∗^ (0.20)

Episode progress	0.00 (0.01)	-0.04^∗∗∗^ (0.01)	-0.05^∗∗∗^ (0.01)
Intercept	0.75^∗∗∗^ (0.13)	0.67^∗∗∗^ (0.12)	0.27^∗∗^ (0.10)

Log likelihood	6.93	14.92	51.87
Akaike Inf. Crit.	8.13	-7.84	-81.75
Bayesian Inf. Crit.	43.79	27.82	-46.09

^∗^*p<0.05; ^∗∗^*p<*0.01; ^∗∗∗^*p<*0.001. *N* = 27*.

### Similarities and Differences Between Pleasure, Interest, and Fear at the Episode Level

Our second research question asks for possible similarities and differences between pleasure, interest, and fear across the 7 self-reported episodes that are nested in the entire event. Among the 38 participants who completed the self-reported episode emotions, six were excluded due to lack of variance (for all episodes, four reported zero fear and two reported maximum pleasure). **Table 6** provides descriptive statistics for the variables.

**Table 6:** Descriptive statistics with mean (M) standard deviation (SD) and standard error of mean (SEM) for self-reported episodic emotions.

	Pleasure			Interest			Fear		
	*M*	*SD*	*SEM*	*M*	*SD*	*SEM*	*M*	*SD*	*SEM*
Episode 1	7.21	2.76	1.17	7.34	2.23	1.19	2.79	2.98	0.45
Episode 2	6.58	2.80	1.07	7.50	2.37	1.22	3.26	2.61	0.53
Episode 3	6.61	2.27	1.07	7.26	2.24	1.18	2.37	2.38	0.38
Episode 4	6.68	2.49	1.08	6.53	2.37	1.06	1.84	1.95	0.30
Episode 5	6.87	2.55	1.11	6.61	2.21	1.07	1.55	1.72	0.25
Episode 6	6.05	3.00	0.98	5.50	2.65	0.89	1.68	1.83	0.27
Episode 7	7.13	2.79	1.16	6.37	2.72	1.03	0.50	0.89	0.08

*Scales for Self-Reported Episodic Emotions are 1–10. Episode refers to the different parts of skiing; (1) At the top, one minute before start, (2) the first part of the descent, (3) before half-way, (4) half-way down the mountain, (5) after half-way, (6) last part of the descent and (7) immediately after they stopped*.

The analysis described in the previous section included a “trend” analysis for pleasure, interest, and fear as the episodes unfolded from the start to the end of the entire event (the episode progress variable). Episode progress turned out to be a significant predictor for the self-reported episode emotions of interest (β = -0.04, *p* < 0.001) and fear (β = -0.049, *p* < 0.001), but not for pleasure (β = 0.001, *p* = 0.93). These results reflect the finding that interest and fear, but not pleasure, declined over the course of the event.

Finally, pairwise within-participant correlations between all pairs of self-reported emotions (fear – interest, fear – pleasure, and pleasure – interest) were calculated. These within-participant correlations were transformed using Fishers Z-transform (Fisher, 1915), averaged and back-transformed. We found that self-reported fear and interest were moderately correlated, *r* = 0.47, *t*(31) = 3.43, *p* = 0.002. In addition, fear and pleasure were negatively related, *r* = -0.18, *t*(31) = -0.80, *p* = 0.43 and pleasure and interest showed a stronger, positive correlation, *r* = 0.52, *t*(31) = 3.06, *p* < 006.

## Discussion

This paper tested a new method of measuring emotions during an extreme sport by capturing and analyzing facially expressed emotions as they unfold moment-by-moment during an intense, outdoor activity. The results show that the participants appeared happier when they had a break than when they were actively skiing. We also found the facially expressed emotions were poor predictors of the self-reported emotions collected immediately after the event. However, facially expressed fear predicted higher levels of self-reported interest, and facially expressed sadness predicted lower levels of self-reported pleasure. There were no apparent beginning or end-effects for facially expressed happiness as a predictor of self-reported happiness.

When the skiing event was separated into 7 successive episodes, the trends for self-reported interest and fear went from higher toward lower levels of intensity. No such decrease was observed for pleasure. Facially expressed fear also predicted lower levels of self-reported pleasure. The correlation between fear and pleasure across the 7 episodes was strongly negative, whereas it was positive between fear and interest. As expected, interest and pleasure correlated positively and significantly.

Our first finding shows that the participants expressed lower levels of happiness during skiing compared to when they stop to take a break. This pattern is consistent with the explanation offered by the flow theory (Csikszentmihalyi, 1999). The theory states that the enjoyment of flow is not consciously experienced, because people in flow are too involved in performing the activity at hand. There are no moment-by-moment experiences present during the flow event, the feelings appear only retrospectively, after the flow state has ended (Seligman, 2002).

Another explanation for our finding is offered by the Functional Wellbeing Approach. In contrast to the flow theory, the FWA suggests that people do have intense feelings during flow, but the experiences are not typically felt as joyful or happy. Rather, what people feel like during flow is interested, immersed and engaged, and these feelings are produced by the step-by-step execution of a challenging activity. The happiness reported afterward, however, is not supposed to be generated by an aggregation of the moment-to-moment feelings during flow, but by a different process altogether. The overall feeling of happiness occurs as the result of having mastered a difficult task. It comes from a positive evaluation of a goal outcome.

The existing literature suggests that both skill development and positive emotions are important motives for extreme sport athletes. But extreme sport is not only positive emotions. Fear is for example the most dominant negative emotion in the present investigation, as it is in other studies (e.g., Brymer and Schweitzer, 2013). As one of the informants in Brymer and Schweitzer (2013) study pointed out, fear forces you to be alert and attentive to potential hazards. However, as Brown and Fraser (2009) argue, there has been a misconception in the literature about fear as a motive of its own for taking part in adventure sports activities. Several studies have described that the participants seek to overcome or master fear (Kerr and Houge Mackenzie, 2012; Langseth, 2015) or a necessary ingredient to produce thrill (Buckley, 2016). Fear is, in other words, not an emotion that the participants seek, but rather one they seek to overcome. They do so by reducing the level of challenge to fit within the frame of their capabilities. In addition to reducing fear, this also creates an arena for learning and development.

Flow theory and FWA disagree on how skills are developed. Flow is characterized by enjoyment and the theory postulates that a precondition for experiencing flow is a match between challenges and skill. The FWA, in contrast, postulates that there is a slight imbalance between experienced challenge and skills (tipping toward more challenging) which is ideal for skill development (Løvoll and Vittersø, 2014). Such an imbalance will lead to growth-oriented action guided by emotions like interest and engagement, not enjoyment. This view is supported by Schwartz and Wrzesniewski (2016) who argue that it is engagement and not pleasure that keeps people working toward difficult goals. Perhaps the most important point in FWA is that such situations, even though they are not pleasant, are still experienced as something worth pursuing for their own sake. Phenomenological they typically feel interesting, absorbing, or challenging.

The results from the self-reported episodes are consistent with the idea that happiness/pleasure and interest are distinct kinds of positive emotions. For instance, facially expressed fear predicted higher self-reported interest and lower self-reported pleasure. Self-reported interest was also positively correlated with fear and both interest and fear were experienced more intensely in the early phases and less intensely in the later phases of the descent. In contrast, self-reported pleasure was negatively correlated with fear and no difference in pleasure intensity was recorded during the event. However, since interest is not included in the FaceReader classification system, no facially expressed data are available to address the issue of how pleasure/happiness and interest might differ. We did not observed much facially expressed happiness during active skiing, though, which indicates that the pleasant kind of feelings are not the dominant experience for high arousal activities.

The FWA argues that whereas the moment-to-moment feelings are responses to the difficulty of ongoing activities, emotional feelings are created in response to an overall evaluation of the goal status for whole events. Moment-to-moment and emotional feelings are therefore responses to two different mental operations. The moment-to-moment feelings are communication about task difficulty while the emotional feelings are communication about goals and values.

In the current study, none of the facially expressed emotions captured during the activity predicted self-reported pleasure or interest. This may have several explanations. First, facially expressed interest is not yet established as a universal facial expression and thus FaceReader, the AFC tool used in this study, does not have the capability to read facially expressed interest.

In a recent article, Campos et al. (2012) found that interest is characterized by lowered eyebrows and raised inner eyebrows. In addition they found a weak correlation between interest and compressed lips and also raised outer eyebrows. Happiness shares none of these characteristics. However, facially expressed sadness is strikingly similar.

In a previous study on the emotional experience of extreme sport, Hetland and Vittersø (2012) found that the participants reported a total absence of sadness. The results in this study, however, show that facially expressed sadness is the second-most prominent emotion expressed during the activity, second only to happiness^1^. A similar result was also found in another recent study where tourists watched tourist commercial films (Hetland et al., 2015). Here the participants expressed more sadness and anger compared to happiness while they watched the films. Afterward, however, they reported high levels of happiness and low levels of sadness.

The results from the current study also show that facially expressed sadness predicts self-reported fear. By itself, such a finding is difficult to explain; yet if we turn to the self-reported episode emotion findings, the results show that interest and fear are positively related. Pleasure and fear, on the other hand, correlate negatively. Seen together, these findings might suggest that facially expressed sadness in some situations can actually be mislabeled interest. Future studies are needed verify the distinct characteristics of facially expressed interest.

Another explanation for the lack of coherence between the self-reported and facially displayed emotions can be that the participants, in fact, are unable to recognize and report their own emotions. This difficulty is called alexithymia, and in a recent study Barlow and his colleges (Barlow et al., 2015) show that people with such difficulties seek extreme sport activities to experience more clear cut emotions. Indeed, Hetland and Vittersø (2012) have shown that the emotional landscape in BASE jumping and skydiving characterized by a few clear and strong emotions, like pleasure and fear – and total absence of others like sadness.

### Limitations and Future Research

This study applied a design with automated facial coding of emotions during high intensity activities. Even though this method opens up new possibilities, it is not without shortcomings. First, this technology was initially developed to function in controlled settings such as a laboratory. Even though the software has become more robust in recent years, the shifting lighting conditions in the field are challenging. For this, and other external factors, a number of participants had to be excluded from the various analyses due to strict criteria for missing data. This led to a limited sample size entering our final analysis, so interpretation of our results should therefore be seen as tentative and as being the result of exploratory rather than a confirmatory nature. The effects found in the current study should therefore serve as an effect-size estimate to inform adequately powered follow-up studies investigating the same or related phenomena.

Also, in order to be able to correctly capture facially expressed emotions, the participants were instructed to ski without goggles. In addition, the camera had to be placed directly in the participants’ field of vision. Although most participants said they quickly habituated to these conditions, these factors might have affected their display of facial expressions.

Moreover, the present study used video-input, and not still images, to analyze facial expressions. Theoretically, film is nothing more than still images displayed in rapid sequence, and FaceReader analyze each frame as a still image. The validity of FaceReaders ability to correctly analyze still images is well established. However, a validity study using video as input would be beneficial in order confirm this validity while using video as input.

In contrast to previous studies of emotional experiences of extreme sport, this study finds that facially expressed sadness is the most prominent moment-to-moment emotion after happiness. Recent studies show that there is a similarity between the facial expressions related to interest and sadness. A possible limitation of the present results is, therefore, that what has been coded as sadness might, in this type of experience, sometimes actually be mislabeled interest.

Relatedly, we lack sufficient knowledge for analyzing emotions that are not “basic” in the Ekman sense of the term. Currently, these are categorized as “Neutral” by the FaceReader, but this “grab-bag” category may have emotional content not captured by the FACS system. Finally, facial expression can, like most other reports of emotions be hidden (Gross and Feldman Barrett, 2011).

In spite of its shortcomings, the FaceReader technology gives us a first peek into how an intense activity like backcountry skiing is experienced as it unfolds – an opportunity that has been unavailable until now. And when it comes to detecting happiness—the major emotion for the present study—the FacerReader seems particularly well equipped.

## Conclusion

The current article provides, to our knowledge, the first study of moment-by-moment emotions during an intense experience like backcountry skiing. It does so by introducing a new way of measuring moment-to-moment emotions in the field by use of small video cameras and software for coding facial expressions. The article argues that the Functional Wellbeing Approach (over the Hedonistic and Gestalt approaches) is best at predicting and understanding how moment-by moment experiences relate to retrospective evaluations. The results show that the skiers express less happiness while they ski, compared to when they stop to take a break. Further, there is no apparent relation between the facially expressed emotions and any of the positive self-reported emotions reported immediately after the trip.

In the end, we may be able to explain why extreme sport athletes report happiness to be their main motivation even though they don’t feel much happiness as long as they are inside the pursuit of the activity. From a functional wellbeing approach, an event can be successfully mastered and thus be remembered as pleasant, even if the subtasks involved are experienced as challenging or even painful. Hence, it seems that memorable events do not develop their emotional meanings from of a series of moment-to-moment feelings—they are generated from something bigger. Much, perhaps, like the happiness of our lives in general.

## Ethics Statement

In the spirit of the Declaration of Helsinki, all of the participants were given full information about the project and what their participation involved. The sample was drawn from a healthy adult population, and all participants signed an informed consent before they were included in the study. The project was approved by the Norwegian Social Science Data Service (license number 32675). In addition institutional and APA ethical standards were followed throughout the work of this study. None of the authors have any conflicting interests.

## Author Contributions

AH is main author of this paper. JV and TD contributed substantially to developing the theory, design, and to writing the manuscript. SW and EK contributed to data collection, preparation for analysis, and writing the method section. MM conducted all statistical analysis and wrote the results section.

## Conflict of Interest Statement

The authors declare that the research was conducted in the absence of any commercial or financial relationships that could be construed as a potential conflict of interest.

## Footnotes

1 Note that the neutral state generated by the AFC software is not seen as an emotion.
